# *Bacillus cereus* Induces Necroptosis in Microglia via the RIPK1/3‐MLKL Pathway

**DOI:** 10.1002/mbo3.70276

**Published:** 2026-03-26

**Authors:** Jing Yang, Bianjin Sun, Huijing Xu, Yangyang Shen, Huili Ye, Qiheng Yuan, Siwen Chen, Meiqin Zheng

**Affiliations:** ^1^ National Clinical Research Center for Ocular Diseases, EyeHospital Wenzhou Medical University Wenzhou China; ^2^ Eye Hospital Wenzhou Medical University Wenzhou China; ^3^ The Second Affiliated Hospital and Yuying Children's Hospital of Wenzhou Medical University Wenzhou China; ^4^ School of Laboratory Medicine and Life Science Wenzhou Medical University Wenzhou China

**Keywords:** *Bacillus cereus*, inflammatory response, microglia, necroptosis

## Abstract

*Bacillus cereus* endophthalmitis is a rapidly progressing intraocular infection that often results in poor visual outcomes due to extensive retinal damage. Microglia are resident innate immune cells in the brain and retina that play critical roles in neurological and ocular diseases. To investigate the functional role and mechanisms of microglia during *B. cereus* infection, we established an in vitro microglial infection model using murine BV2 and human HMC3 cells. *B. cereus* infection reduced microglial viability and induced membrane rupture. Transcriptome analysis revealed enrichment of inflammatory and cell death. Flow cytometric screening identified that the RIPK1 inhibitor Nec‐1 rescued cell death. Mechanistically, *B. cereus* increased phosphorylation of RIPK3 and MLKL, which was abolished by Nec‐1. Moreover, Nec‐1 suppressed *B. cereus*‐induced secretion of IL‐1β, IL‐6, and TNF‐α. In summary, this study demonstrates that *B. cereus* induces microglial necroptosis by activating the RIPK3/MLKL pathway, providing a new mechanism for neuroinflammation caused by related infections.

## Graphical abstract

*Bacillus cereus* induces necroptosis in murine/human microglia via the RIPK1/3‐MLKL pathway, triggering inflammatory cytokine release—uncovering a new pathogenic mechanism for its severe ocular infection.

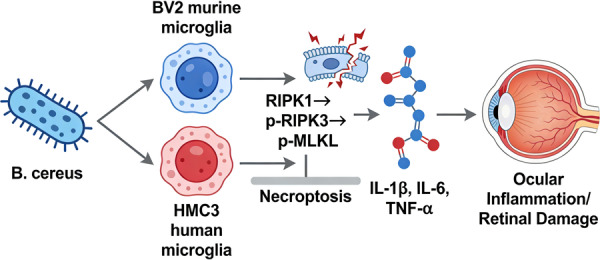

## 1 Introduction

The symptoms and disease outcomes of different types of endophthalmitis are comparable and range from red, inflamed eyes to extreme intraocular pain and vision loss, irrespective of the etiology of infection (Das et al. 2023). Endophthalmitis is more commonly caused by bacteria than other organisms (Parvizi et al. 2020; Das and Sharma 2018). *Bacillus* intraocular infection progresses very rapidly. *Bacillus cereus* is common in the environment, an important human foodborne pathogen, and the second most common pathogen that causes endophthalmitis (Hong et al. 2016). *B. cereus* causes gastrointestinal diseases (Jovanovic et al. 2021) or a variety of systemic infections (Ehling‐Schulz et al. 2019; Glasset et al. 2018), such as endophthalmitis and endocarditis. *B. cereus* endophthalmitis progresses rapidly and deteriorates sharply, resulting in severe destruction and lysis of tissue; therefore, the window for successful therapeutic intervention is relatively short (Astley et al. 2016; Mursalin et al. 2020; Callegan et al. 2005). Patients who have a poor prognosis ultimately require eye enucleation even after vitrectomy or antibiotic treatment (Callegan et al. 2002; Relhan et al. 2016; Zheng et al. 2024). *B. cereus* has a rapid intraocular reproduction rate and produces many virulence factors that affect the host (Callegan et al. 2002). *B. cereus* is highly virulent and induces gastrointestinal diseases (Relhan et al. 2016), cell apoptosis (Glasset et al. 2021), severe inflammatory responses (Moyer et al. 2008), and immune evasion. *B. cereus* can kill corneal stromal cells in vitro within 6 h (Mursalin et al. 2020). Early in the infected mouse model, many inflammatory cells infiltrate the vitreous and local cytokine levels are significantly elevated, with almost complete loss of retinal function at 12 h of infection (Mursalin et al. 2020; Moyer et al. 2008; Xiao et al. 2023).

Microglia are macrophages in the brain and retina that have immunomodulatory and phagocytic functions and play important roles in maintaining homeostasis (Nayak et al. 2014; Chen et al. 2019). In the healthy central nervous system, resting microglia provide trophic support and constantly and sensitively monitor pathological changes in the local microenvironment (Prinz et al. 2019; Colonna and Butovsky 2017; Fattorelli et al. 2021). Under pathological conditions, damaged neurons release signals to recruit microglia rapidly in the lesional area, induce microglial proliferation and infiltration, and phagocytose necrotic neurons and deleterious factors. Microglial activation usually induces proneuronal growth or the secretion of cytotoxic factors to conversely aggravate injury (Silverman and Wong 2018; Huang et al. 2018). In acute retinal injury mouse models, microglia undergo necroptosis and produce large amounts of proinflammatory cytokines, which mediate neural injuries and neurodegenerative diseases (Huang et al. 2018; Karlstetter et al. 2015; Schuetz and Thanos 2004). In host defense against microbial pathogens, innate immunity is the first line of defense against invading pathogens (Callegan et al. 2005, 2002). Various retinal cells, such as microglia (Rashid et al. 2019; Li et al. 2019; Huang et al. 2020) and Müller glia, express Toll‐like receptors (TLRs) and orchestrate retinal innate responses in bacterial endophthalmitis (Wang and Wong 2014). However, the specific response of microglia to B. cereus infection and the molecular mechanisms governing their fate remain largely unexplored.

Necroptosis can form a necrosome (Xu et al. 2021), which induces cell membrane rupture and the release of damage‐associated molecular pattern (DAMP) molecules, aggravating inflammatory responses and tissue damage (Weinlich et al. 2017; Yuan et al. 2019; Qiu et al. 2023; Shi et al. 2019). For example, muscle cells in idiopathic inflammatory myopathy (IIM) undergo necroptosis and promote muscle inflammation and dysfunction (Shi et al. 2021). The main type of cell death under physiological conditions is apoptosis, whereas pathogen infection often induces necroptosis or pyroptosis, which activate the immune system and trigger inflammation (Bertheloot et al. 2021; Wu et al. 2020). Increasing evidence suggests the pivotal role of necroptosis in inflammation. Given the pronounced inflammation and tissue destruction characteristic of *B. cereus* endophthalmitis, necroptosis may represent a key mechanism of retinal cell death and inflammation.

In this study, we investigated the functional role and molecular mechanisms of microglial response to B. cereus infection. Specifically, we examined whether B. cereus infection triggers necroptosis in microglia and explored the associated inflammatory responses. Our findings may provide new insights into the pathogenesis of bacterial endophthalmitis and identify potential therapeutic targets for preserving retinal function.

## 2 Materials and Methods

### 2.1 Bacterial Culture

Clinical isolates of *B. cereus* strain Bc7 (from patients with vision loss) were grown overnight in LB medium at 37°C aerobically. The next day, bacteria were passaged 1:10 in fresh LB medium for 3–4 h to logarithmic phase.

### 2.2 Cell Culture and Treatment

BV2 microglial cells (China Typical Culture Preservation Center, Wuhan) were cultured in high‐glucose Dulbecco's modified Eagle's medium (DMEM) complete medium (Thermo Fisher Scientific, USA). HMC3 cells (Procell system, Wuhan) were maintained in MEM supplemented with 10% fetal bovine serum and 1% penicillin/streptomycin. All cells were incubated at 37°C with 5% CO₂until confluent.

To assess time‐ and dose‐dependent cytotoxicity, BV2 and HMC3 cells were exposed to Bc7 at different multiplicities of infection (MOIs: 0, 0.5, 1, 2, 4) for 1‐4 h, with viability quantified by Cell Counting Kit‐8 (CCK‐8) assay (MCE, USA). For pretreatment, cells were incubated with 5–50 μM Z‐VAD‐FMK (ZVAD, apoptosis inhibitor), 2.5–10 μM Disulfiram (DSF, pyroptosis inhibitor), or 100–300 μM Necrostatin‐1 (Nec‐1, necroptosis inhibitor) for 1 h prior to Bc7 infection.

### 2.3 Cell Viability Assay

BV2/HMC3 cells were seeded in 96‐well plates (1×10⁵ cells/mL, 100 μL/well) and treated with Bc7 at aforementioned MOIs for 1–4 h. After treatment, 10 μL of CCK‐8 reagent was added to each well (mixed with the existing 100 μL medium), and plates were incubated for an additional 2 h at 37°C. Absorbance at 450 nm was measured using a microplate reader (Molecular Devices, USA), with viability calculated relative to untreated controls.

### 2.4 Cell Density and Morphology Analysis

BV2/HMC3 cells were seeded in 6‐well plates (5×10⁵ cells/mL) and co‐cultured with Bc7 (MOIs: 0, 0.5, 1, 2, 4) for 1–4 h. Cell density and morphology were observed and photographed via inverted phase‐contrast fluorescence microscope (Zeiss).

For detailed observation, cells grown on coverslips were infected with Bc7 (MOI = 2, 2 h), then subjected to Gram (for *B. cereus*) and Wright's (for microglia) staining, followed by oil immersion microscopy. Gram staining: crystal violet (1 min), iodine (1 min), 95% ethanol (1 min), safranin (1 min). Wright's staining: air‐dry, Solution A (1 min), Solution B (3–5 min), rinse and air‐dry.

### 2.5 Flow Cytometric Analysis

BV2 cells were seeded in 6‐well plates (5×10^5^cells/mL) and pretreated with 100/200/300 μM Nec‐1 for 1 h (per Ref (Bertheloot et al. 2021).), then infected with Bc7 (MOI = 2) for 2 h. Groups: (1) control; (2) Bc7‐infected (BV2 + Bc7); (3) 100 μM Nec‐1 (BV2 + 100 μM Nec‐1 alone/BV2 + 100 μM Nec‐1 + Bc7 infected); (4) 200 μM Nec‐1 (alone/infected); (5) 300 μM Nec‐1 (alone/infected). Z‐VAD‐FMK/DSF groups followed the same strategy.

Apoptosis/necrosis were detected via Annexin V‐FITC Kit (Biyun Tian). Cells were washed with PBS, digested with 0.25% trypsin, centrifuged (1000*g*, 5 min), resuspended in 195 μL binding buffer, and stained with 5 μL Annexin V‐FITC and 10 μL PI for 20 min at room temperature in the dark before analysis by flow cytometry (Beckman Coulter, USA). Late apoptosis/necrosis were quantified by the Annexin V^+^/PI^+^ cell population.

### 2.6 RNA‐seq and Analysis

Total RNA was extracted via TRIzol (Invitrogen), with quality verified by 1% agarose gel electrophoresis and Nanodrop spectrophotometry. cDNA libraries were constructed using NEBNext Ultra II RNA/Directional RNA Library Prep Kits (NEB), size‐selected (400–500 bp) via AMPure XP beads, purified, and sequenced on an Illumina sequencer in PE150 mode. Low‐quality reads (average Q20 < threshold) were filtered by Cutadapt, clean reads aligned to the reference genome via HISAT2, and gene expression normalized by FPKM (Fragments Per Kilobase of transcript per Million mapped reads), with FPKM > 1 considered positive expression. Differentially expressed genes (DEGs) were identified by DESeq with criteria of |log₂FoldChange | > 1 and *p* < 0.05. Principal component analysis (PCA), Gene Ontology/Kyoto Encyclopedia of Genes and Genomes (GO/KEGG) enrichment analyses, and hierarchical clustering (Euclidean distance, Complete Linkage) were performed via DESeq and Pheatmap packages in R, with *p* < 0.05 considered statistically significant.

### 2.7 Western Blotting

Treated BV2 and HMC3 cells were lysed in radioimmunoprecipitation assay lysis buffer (containing protease and phosphatase inhibitors) and incubated on ice for 30 min, homogenized, and centrifuged (12,000*g*, 15 min, 4°C) to collect the supernatant. Protein concentration was quantified by BCA assay (Thermo Fisher Scientific) and normalized to 2 μg/μL. Equal amounts of protein (30 μg per lane) were 10% sodium dodecyl sulfate‐polyacrylamide gel electrophoresis gel electrophoresis and transferred to polyvinylidene difluoride (PVDF) membranes, blocked with 5% skimmed milk in TBST buffer for 2 h at room temperature, and incubated with primary antibodies (against RIPK3, p‐RIPK3 1:1000; MLKL, p‐MLKL 1:500; β‐actin 1:20,000) overnight at 4°C (Cell Signaling Technology, USA). After three washes with TBST buffer (5 min each), membranes were incubated with HRP‐conjugated secondary antibodies (1:5000) for 1 h at room temperature. Protein bands were visualized using the Extremely Ultrasensitive ECL Chemiluminescence Kit (Boster Biological Technology, Wuhan, China) and quantified via ImageJ software (National Institutes of Health, USA), with β‐actin as the internal reference.

### 2.8 Enzyme‐Linked Immunosorbent Assay (ELISA)

Supernatants from Bc7‐infected BV2/HMC3 cells (MOI = 2, 2 h). Interleukin (IL)‐1β, IL‐6, and tumor necrosis factor‐α (TNF‐α) concentrations were measured via ELISA kits (JONLNBIO, China) per manufacturer's instructions. By double‐antibody sandwich method: supernatants/standards/biotin‐labeled antibodies (37°C, 1 h), followed by HRP‐conjugate (37°C, 30 min). After washing, TMB was added (10 min, room temperature, dark), reaction terminated with stop solution, and absorbance measured at 450 nm to calculate concentrations via standard curve.

### 2.9 Quantitative Real‐time Polymerase Chain Reaction (RT‐qPCR)

Total RNA was reverse‐transcribed into cDNA via PrimeScript™ RT reagent Kit (Takara, with gDNA eraser). RT‐qPCR was performed via TB Green® Premix Ex Taq™ II (Takara) on QuantStudio 6 system. Primer sequences are in Supporting Information: Table 1. Reactions were run in triplicate, and target gene (IL‐1β, IL‐6, TNF‐α, Ripk3, and Nos2) expression was normalized to GAPDH via 2^−ΔΔCt^ method.

### 2.10 Statistical Analysis

Data were expressed as mean ± standard error of mean (SEM) and analyzed via GraphPad Prism 8.3.0. Differences were compared by one‐way analysis of variance (ANOVA) (Tukey's post hoc) or *t*‐test. *p* < 0.05 was statistically significant.

## 3 Results

### 3.1 Bc7 Infection Reduces Viability and Alters Morphology of BV2 and HMC3 Microglia

**Figure 1:**
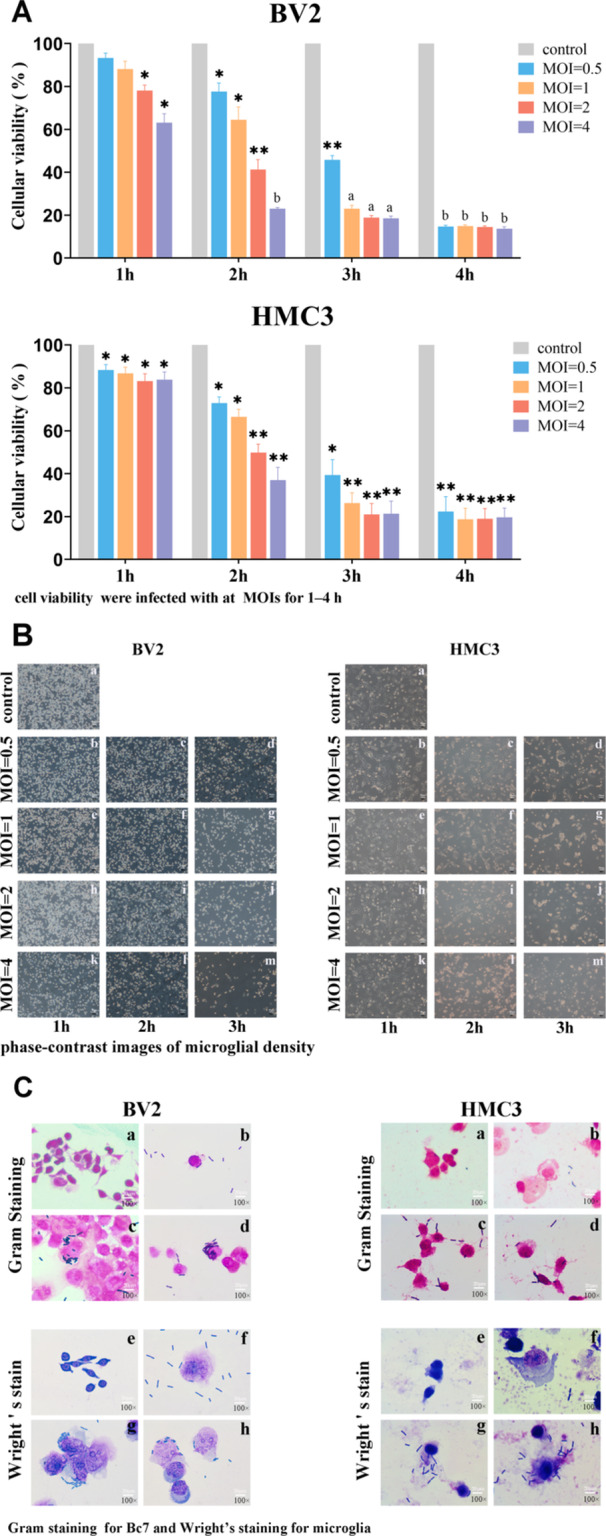
Bc7 infection reduces microglial viability and alters cell morphology. (A) BV2 and HMC3 cells were infected with Bc7 at the indicated MOIs for 1–4 h. Cell viability was measured by CCK‐8 assay and normalized to uninfected controls (MOI = 0). Data are shown as mean ± SEM from three independent experiments. *p < 0.05, **p < 0.01, ^a^ *p* < 0.001, ^b^ *p* < 0.0001 versus control. (B) Representative phase‐contrast images of microglial density under indicated conditions. Scale bar = 50 μm. (C) Gram staining (a–d) and Wright's staining (e–h) of microglia infected with Bc7 at MOI = 2 for 2 h. (a, e) Uninfected controls; (b–d, f–h) infected cells. Arrowheads indicate Bc7 in proximity to or in contact with microglia. Infected cells show membrane rupture and cytoplasmic swelling. Scale bar = 20 μm. CCK‐8, Cell Counting Kit‐8; MOI, multiplicity of infection; SEM, standard error of mean.

To investigate the effect of *B. cereus* infection on microglia, murine BV2 and human HMC3 cells were infected with Bc7 at MOIs of 0, 0.5, 1, 2, and 4 for 1, 2, 3, and 4 h. Cell viability was measured by CCK‐8 assay. Bc7 infection reduced cell viability in a time‐ and dose‐dependent manner (Figure 1A). At MOI = 2, viability of both BV2 and HMC3 cells decreased to approximately 80% at 1 h and 40% at 2 h post‐infection (*p* < 0.05 vs. control). The reduction became more pronounced at higher MOIs and longer infection times. Consistent with the viability data, microglial density observed under phase‐contrast microscopy declined progressively with increasing MOI and infection duration (Figure 1B). To visualize bacterial interaction and morphological changes, Gram and Wright's staining were performed. At 2 h postinfection (MOI = 2), Bc7 was observed in proximity to or in contact with microglia. Infected cells exhibited membrane rupture and increased cytoplasmic volume, paler cytoplasmic staining, and blurred cytoplasmic boundaries (Figure 1C). Based on these results and previous reports (Wang et al. 2022a), infection for 1 h and 2 h at MOI = 2 was selected for subsequent experiments: 1 h‐infected cells (≈80% viable) for RNA extraction, and 2 h‐infected cells (≈40% viable) for protein extraction, to obtain optimal yields.

### 3.2 Transcriptional Profiling of Bc7‐infected BV2 Microglia

**Figure 2:**
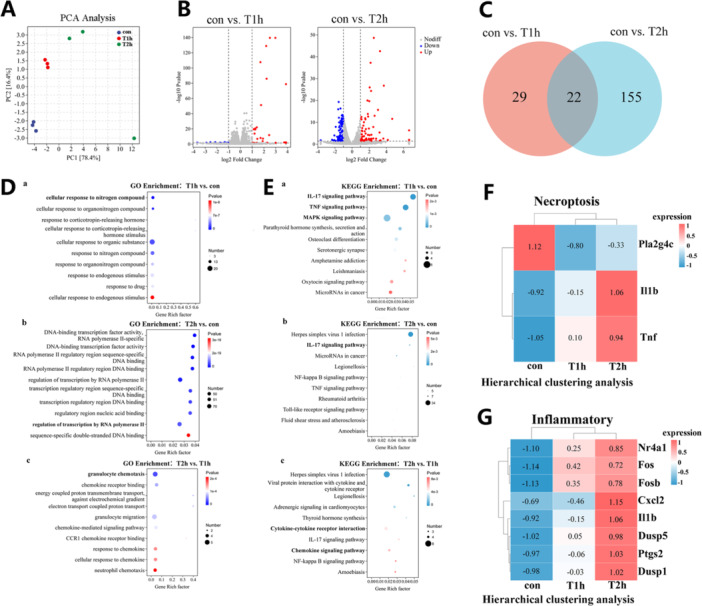
RNA sequencing analysis of Bc7‐infected BV2 cells. (A) PCA of RNA‐seq samples from uninfected BV2 cells (Con) and cells infected with Bc7 at MOI = 2 for 1 h (T1h) or 2 h (T2h). *n* = 3 biological replicates per condition. (B) Volcano plots showing DEGs in T1h versus Con and T2h versus Con. Red: upregulated (| log₂FC | ≥ 1, adjusted *p* < 0.05); blue: downregulated. (C) Venn diagram of DEGs identified at T1h and T2h. (D) Top 10 enriched GO biological process terms for DEGs in the indicated comparisons. (E) Top 10 enriched KEGG pathways for DEGs in the indicated comparisons. (F, G) Heatmaps of DEGs related to necroptosis (F) and inflammatory pathways (G). Color scale indicates row‐scaled expression levels. DEGs, differentially expressed genes; KEGG, Kyoto Encyclopedia of Genes and Genomes; MOI, multiplicity of infection.

To assess Bc7‐induced transcriptional changes, BV2 cells were infected with Bc7 at MOI = 2 for 1 h (T1h) or 2 h (T2h), and subjected to RNA‐seq. Uninfected cells served as controls (Con). PCA showed distinct clustering of Con, T1h, and T2h samples, with T1h and T2h profiles closer to each other than to Con (Figure 2A). Differentially expressed genes (DEGs) were identified using |log₂FC | ≥ 1 and adjuted *p* < 0.05. Compared to Con, 51 DEGs were detected at T1h and 177 at T2h (Figure 2B,C). Among these, 22 DEGs (43.1% of T1h DEGs) were shared between the two time points, 20 of which (90.9%) were upregulated (Supporting Information: Table 2). These results suggest that differences in *B. cereus* infection‐induced gene expression increase over time. GO enrichment analysis revealed that T1h DEGs were associated with responses to nitrogen compounds and endogenous stimuli. T2h DEGs were enriched in regulatory DNA binding terms (Figure 2D). KEGG pathway analysis showed that T1h DEGs were enriched in IL‐17, TNF, and MAPK signaling pathways. Comparison of T2h versus T1h identified enrichment in cytokine–receptor interaction, chemokine signaling, and IL‐17 signaling (Figure 2E). To examine pathways related to cell death, we analyzed DEGs involved in necroptosis and inflammatory responses. Hierarchical clustering of necroptosis‐related DEGs (Figure 2F) and inflammatory pathway DEGs (Figure 2G) showed time‐dependent expression changes, including upregulation of Il1b, Tnf, Cxcl2, and Dusp1.

### 3.3 *Bacillus* Induces Necroptosis in Microglia

**Figure 3:**
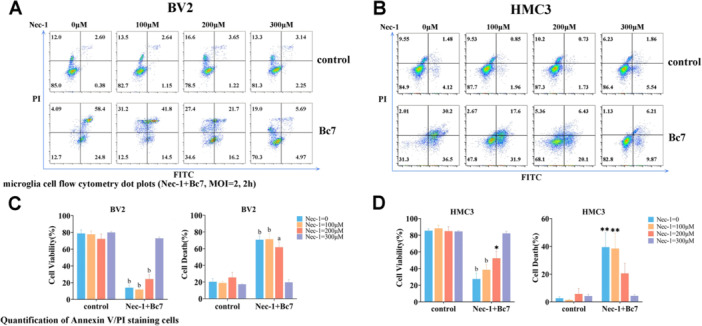
Nec‐1 protects microglia from Bc7‐induced cell death in a dose‐dependent manner. (A, B) BV2 (A) and HMC3 (B) cells were infected with Bc7 (MOI = 2, 2 h) with or without Nec‐1 pretreatment at indicated concentrations. Cell death was analyzed by Annexin V/PI staining and flow cytometry. Representative dot plots are shown. (C, D) Quantification of viable, late apoptotic, and necrotic cells from (A) and (B). Data are shown as mean ± SEM from three independent experiments. *p < 0.05, **p < 0.01, ^a^ *p* < 0.001, ^b^ *p* < 0.0001 versus the corresponding drug concentration gradient in the control. FITC, fluorescein isothiocyanate; MOI, multiplicity of infection; SEM, standard error of mean.

To explore the mode of microglial death induced by Bc7, we hypothesized that infected cells would trigger programmed death through certain pathogen–host interaction signals, including apoptosis, pyroptosis, and necroptosis. Pretreatment with the above cell death inhibitors allowed us to preliminarily screen for the mode of cell death in Bc7‐infected microglia via flow cytometry. Additionally, we examined the survival, late apoptosis, and necrosis of BV2 and HMC3 cells at MOI = 2, incubated for 2 h, three cases of the two microglias were repeated and analyzed. Pretreatment with Nec‐1, a RIPK1 inhibitor, significantly reduced Bc7‐induced cell death in both BV2 and HMC3 cells (Figure 3A,B). In contrast, ZVAD (pan‐caspase inhibitor) and DSF (GSDMD pore formation inhibitor) showed no significant protective effect (Supporting Information: Figures 1A–D and 2A–D). Quantitative analysis revealed that Nec‐1 treatment increased the proportion of viable cells and decreased late apoptotic/necrotic populations in a dose‐dependent manner, with maximal effect at 300 μM (Figure 3C,D). These results indicate that Bc7 infection triggers necroptosis in microglia, which is dependent on RIPK activity.

### 3.4 Bc7 Induces RIPK3/MLKL Phosphorylation and Necroptosis via RIPK1 Kinase Activity

**Figure 4:**
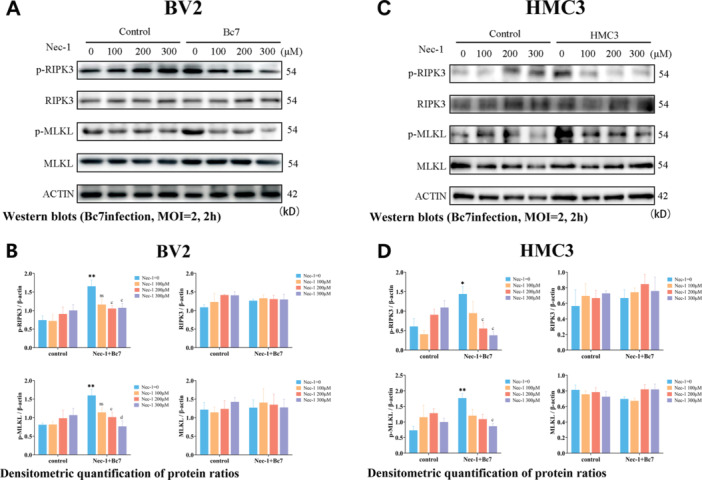
Bc7 induces RIPK3/MLKL phosphorylation in microglia. (A, C) Representative Western blots of p‐RIPK3, total RIPK3, p‐MLKL, and total MLKL in BV2 (A) and HMC3 (C) cells infected with Bc7 (MOI = 2, 2 h) with or without Nec‐1 pretreatment at indicated concentrations. β‐actin was used as loading control. (B, D) Densitometric quantification of p‐RIPK3/RIPK3 and p‐MLKL/MLKL ratios from (A) and (B). Data are shown as mean ± SEM from three independent experiments. *p < 0.05, **p < 0.01 versus control Nec‐1 = 0; ^c^ *p* < 0.05, ^d^ *p* < 0.01, ^e^ *p* < 0.001 versus Nec‐1 + Bc7 group Nec‐1 = 0. MOI, multiplicity of infection; SEM, standard error of mean.

Nec‐1 is an inhibitory drug that specifically targets RIPK1 proteins. To determine whether Bc7 infection activates the necroptosis executioners RIPK3 and MLKL, BV2 and HMC3 cells were infected with Bc7 (MOI = 2, 2 h) with or without Nec‐1 pretreatment. Phosphorylated and total protein levels of RIPK3 and MLKL were examined by Western blot. In BV2 cells, Bc7 infection significantly increased the levels of p‐RIPK3 and p‐MLKL without affecting total RIPK3 or MLKL (Figure 4A,B). This phosphorylation was abolished by Nec‐1 pretreatment in a dose‐dependent manner, with significant reduction starting at 200 μM (*p* < 0.05) and maximal inhibition at 300 μM (*p* < 0.01). Similar results were observed in HMC3 cells (Figure 4C,D). Consistent with the cell death rescue data (Figure 3), these results demonstrate that Bc7 infection triggers RIPK3/MLKL phosphorylation and necroptosis through a Nec‐1‐sensitive, RIPK1 kinase activity‐dependent mechanism.

### 3.5 Nec‐1 Suppresses Bc7‐induced Inflammatory Cytokine Production in Microglia

**Figure 5:**
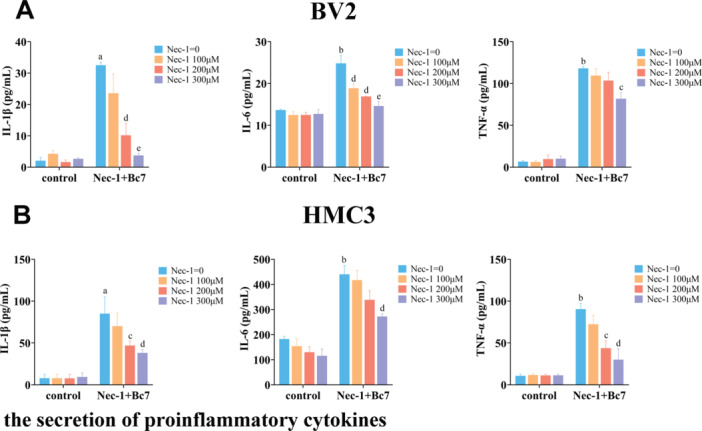
RIPK1 inhibition reduces Bc7‐induced proinflammatory cytokine secretion in microglia. (A, B) BV2 (A) and HMC3 (B) cells were infected with Bc7 (MOI = 2, 2 h) with or without Nec‐1 pretreatment at indicated concentrations. Secreted IL‐1β, IL‐6, and TNF‐α in culture supernatants were measured by ELISA. Data are shown as mean ± SEM from three independent experiments. **p < 0.01, ^a^ *p* < 0.001, ^b^ *p* < 0.0001 versus control Nec‐1 = 0; ^c^ *p* < 0.05, ^d^ *p* < 0.01, ^e^ *p* < 0.001 versus Nec‐1 + Bc7 group, Nec‐1 = 0. ELISA, enzyme‐linked immunosorbent assay; IL, interleukin; MOI, multiplicity of infection; SEM, standard error of mean; TNF‐α, tumor necrosis factor‐α.

Neuroinflammation is a well‐documented consequence of microglial necroptosis in various neurological conditions. To determine whether necroptosis contributes to the inflammatory response induced by Bc7 infection, we measured the secretion of proinflammatory cytokines in Bc7‐infected microglia with or without Nec‐1 pretreatment. BV2 and HMC3 cells were infected with Bc7 (MOI = 2, 2 h) in the presence of increasing concentrations of Nec‐1 (100–300 μM). Culture supernatants were collected and analyzed by ELISA. Bc7 infection significantly increased the secretion of IL‐1β, IL‐6, and TNF‐α in both BV2 (Figure 5A) and HMC3 (Figure 5B) cells. This elevation was attenuated by Nec‐1 in a dose‐dependent manner, with significant inhibition observed at 200 μM and 300 μM. These results indicate that RIPK1 kinase activity mediates not only Bc7‐induced necroptosis but also the associated proinflammatory cytokine response in microglia.

## 4 Discussion

Ocular infection caused by *B. cereus* is a devastating condition characterized by rapid intraocular proliferation (Enosi Tuipulotu et al. 2021), antibiotic insensitivity, and fulminant inflammation, often leading to irreversible retinal damage and subsequent vision loss (Astley et al. 2016; Relhan et al. 2016; Glasset et al. 2021; Callegan et al. 2006; Liu et al. 2022; Coburn et al. 2018). As the primary resident innate immune sentinels in the retina, microglia maintain ocular homeostasis and initiate local immune responses upon microbial challenge (He et al. 2021). However, the eventual death of these activated immune cells does not necessarily resolve inflammation (Deng et al. 2021; Paget et al. 2023). Necroptosis—a regulated, pro‐inflammatory form of programmed cell death distinct from passive necrosis (Newton and Gitlin 2022; Park et al. 2023)—has emerged as a potential mechanism linking cell death to exacerbated inflammatory damage in *B. cereus* endophthalmitis.

*B. cereus* infection exerted a rapid and time‐dependent cytotoxic effect on microglia. Within 2 h of infection at an MOI of 2, cell viability was reduced by approximately 50%, accompanied by hallmarks of necrotic morphology—cytoplasmic swelling and membrane blebbing. This cytotoxic phenotype was paralleled by a robust pro‐inflammatory response, with marked upregulation of IL‐1β, IL‐6, and TNF‐α at both transcriptional and protein levels. The convergence of lytic cell death kinetics and a hyper‐inflammatory signature strongly suggested the involvement of a regulated, pro‐inflammatory necrotic pathway—prompting a focused investigation into necroptosis.

To gain broader insight, we conducted transcriptomic analysis, which confirmed a robust early inflammatory response (Yang et al. 2024) but did not pinpoint a specific death pathway at the transcriptional level. Given the pro‐inflammatory nature of the observed cell lysis, we specifically investigated necroptosis and directly analyzed the activation of its core DEGs. These DEGs were significantly upregulated in cytokine signaling and cell necroptosis at early infection time points (1 h and 2 h). To definitively identify the cell death pathways, we first employed flow cytometry using specific inhibitors against key programmed cell death modalities. Pretreatment with the necroptosis inhibitor Nec‐1 (targeting RIPK1) significantly rescued microglial viability and reduced the Annexin V^+^/PI^+^ cell population during *B. cereus* infection. In contrast, inhibitors of apoptosis (Z‐VAD) and pyroptosis (DSF) showed no protective effect, confirming necroptosis as the essential and predominant death pathway. This was corroborated at the molecular level. Bc7 infection induced phosphorylation of RIPK3 and MLKL, and this activation was completely abolished by Nec‐1 pretreatment, confirming the causal role of the RIPK1/RIPK3/MLKL axis. To link this death pathway to the inflammatory phenotype, we assessed cytokine secretion. Nec‐1 significantly attenuated the release of IL‐1β, IL‐6, and TNF‐α, indicating that a substantial portion of the inflammatory response is downstream of, and dependent on, necroptosis execution. In summary, our data trace a coherent pathogenic sequence: *B. cereus* induces microglial necroptosis, and molecular analysis confirms activation of the core RIPK3/MLKL signaling axis and its functional role in triggering and amplifying inflammatory responses.

The present in vitro findings are generally consistent with previous studies reporting that pathogenic bacteria trigger cell death or inflammatory responses in retinal cells such as RPE. For instance, prior work has documented that *B. cereus* induces RPE cytotoxicity, and increases the rate of cell death, which contribute to the deleterious effects of *B. cereus* infection on RPE (the primary cells of the blood‐retina barrier) barrier function (Moyer et al. 2008). Transcriptomic analysis revealed that *B. cereus* infection triggered inflammatory (Yang et al. 2024) and cell death pathways in microglia, including IL‐17, TNF‐α, and MAPK signaling (Shan et al. 2018; Varfolomeev and Vucic 2018). These observations are consistent with the well‐established role of TNF signaling in promoting both inflammation and regulated necrosis (Noviello et al. 2021; Huyghe et al. 2023; Sun et al. 2015; Wang et al. 2022b). The concurrent induction of necroptotic signaling and inflammatory cytokine release in our study further supports that microglial death programs and inflammatory responses are tightly coupled during *B. cereus* infection. This pathogen–host interaction profile is distinct from that reported in other viral contexts, such as SARS‐CoV‐2‐induced microglial apoptosis (Jeong et al. 2022), and reinforces the emerging view that different pathogens can engage distinct programmed cell death modalities in the same cell type. By coupling transcriptomic screening with functional inhibitor assays and molecular validation, our study provides the first evidence, to our knowledge, that necroptosis is the principal cell death pathway activated by *B. cereus* in microglia, and that this pathway reinforces the cytotoxic and inflammatory potential of *B. cereus* in ocular innate immune cells.

Our study has several limitations. This in vitro study used immortalized microglial cell lines that cannot fully recapitulate the retinal microenvironment or systemic immune influences present in vivo. Future studies employing animal models of *B. cereus* endophthalmitis are essential to validate the role of microglial necroptosis in disease progression. Additionally, although necroptosis was confirmed in our system, the upstream bacterial ligands and host sensors that initiate this pathway remain unclear. Future work should explore whether *B. cereus* toxins or pattern recognition receptors activate the necroptosis pathway. Our transcriptomic data showed enhanced intercellular signaling and neutrophil recruitment, but their functional roles and crosstalk with retinal cells were not explored. Nevertheless, our findings have translational potential. Necroptosis has dual roles: it drives inflammation and tissue damage, but also contributes to host defense (Mohammed et al. 2021; Yan et al. 2022; Yu et al. 2013; Lin et al. 2016). In *B. cereus* endophthalmitis, where infection progresses faster than adaptive immunity, the inflammatory cost of necroptosis outweighs its protective value. Unlike chronic microgliosis in neurodegeneration (Subhramanyam et al. 2019; DeMaio et al. 2022), *B. cereus*‐induced rapid microglial death provides a unique therapeutic window. Thus, targeting the necroptosis pathway may serve as a host‐directed adjunct strategy for antibiotic‐refractory cases. Transcriptionally enhanced signaling and neutrophil recruitment may provide targets for cell‐type‐specific therapies. In summary, we have identified necroptosis as the principal cell death pathway in *B. cereus*‐infected microglia and linked it to amplified inflammatory responses. This provides a novel cellular mechanism contributing to the rapid tissue destruction in ocular infections. Targeting the necroptosis pathway may therefore represent a promising therapeutic strategy to attenuate excessive inflammation and preserve vision in this devastating disease.

## Author Contributions

**Jing Yang:** conceptualization, methodology, investigation, formal analysis, data curation, writing – review and editing. **Bianjin Sun:** methodology, formal analysis, data curation, writing – review and editing. **Huijing Xu:** conceptualization, methodology, writing – review and editing. **Yangyang Shen:** resources, writing – review and editing. **Huili Ye:** investigation, resources, writing – review and editing. **Qiheng Yuan:** resources, writing – review and editing. **Siwen Chen:** resources, writing – review and editing. **Meiqin Zheng:** conceptualization, methodology, supervision, project administration, funding acquisition, writing – review and editing. All authors have read and approved the final version of the manuscript.

## Ethics Statement

The authors have nothing to report.

## Conflicts of Interest

The authors declare no conflicts of interest.

## Supporting information

supmat.
