# Supplementary material for: Bacillus cereus Induces Necroptosis in Microglia via the RIPK1/3‐MLKL Pathway
Source: Microbiologyopen. 2026 Mar 26;15(2):e70276. doi: 10.1002/mbo3.70276 (PMC13140653; doi:10.1002/mbo3.70276)
Supplement: Supplementary file 1 — supmat. [file MBO3-15-e70276-s001.docx]

**Supplementary Tables and Figures**

**Supplementary Table. 1 RT-qPCR Primer**

| Gnens |  | Primer sequence（5’-3’） |
| --- | --- | --- |
| TNF-α(Mouse) | forward primer | CCACCACGCTCTTCTGTCTACTG |
|  | reverse primer | GGGCTACAGGCTTGTCACTCG |
| IL-6(Mouse) | forward primer | GAAATGCCACCTTTTGACAGTG |
|  | reverse primer | TGGATGCTCTCATCAGGACAG |
| IL-1β(Mouse) | forward primer | CTGCAAGAGACTTCCATCCAG |
|  | reverse primer | AGTGGTATAGACAGGTCTGTTGG |
| IL-10(Mouse) | forward primer | AGCCTTATCGGAAATGATCCAGT |
|  | reverse primer | GGCCTTGTAGACACCTTGGT |
| Ripk1(Mouse) | forward primer | GAAGACAGACCTAGACAGCGG |
|  | reverse primer | CCAGTAGCTTCACCACTCGAC |
| Ripk3(Mouse) | forward primer | TCTGTCAAGTTATGGCCTACTGG |
|  | reverse primer | GGAACACGACTCCGAACCC |
| Nos2(Mouse) | forward primer | GGAGTGACGGCAAACATGACT |
|  | reverse primer | TCGATGCACAACTGGGTGAAC |
| Arg1(Mouse) | forward primer | CTCCAAGCCAAAGTCCTTAGAG |
|  | reverse primer | AGGAGCTGTCATTAGGGACATC |
| GAPDH(Mouse) | forward primer | TGACCTCAACTACATGGTCTACA |
|  | reverse primer | CTTCCCATTCTCGGCCTTG |

**Supplementary Table. 2 These genes were upregulated at both 1h and 2h**


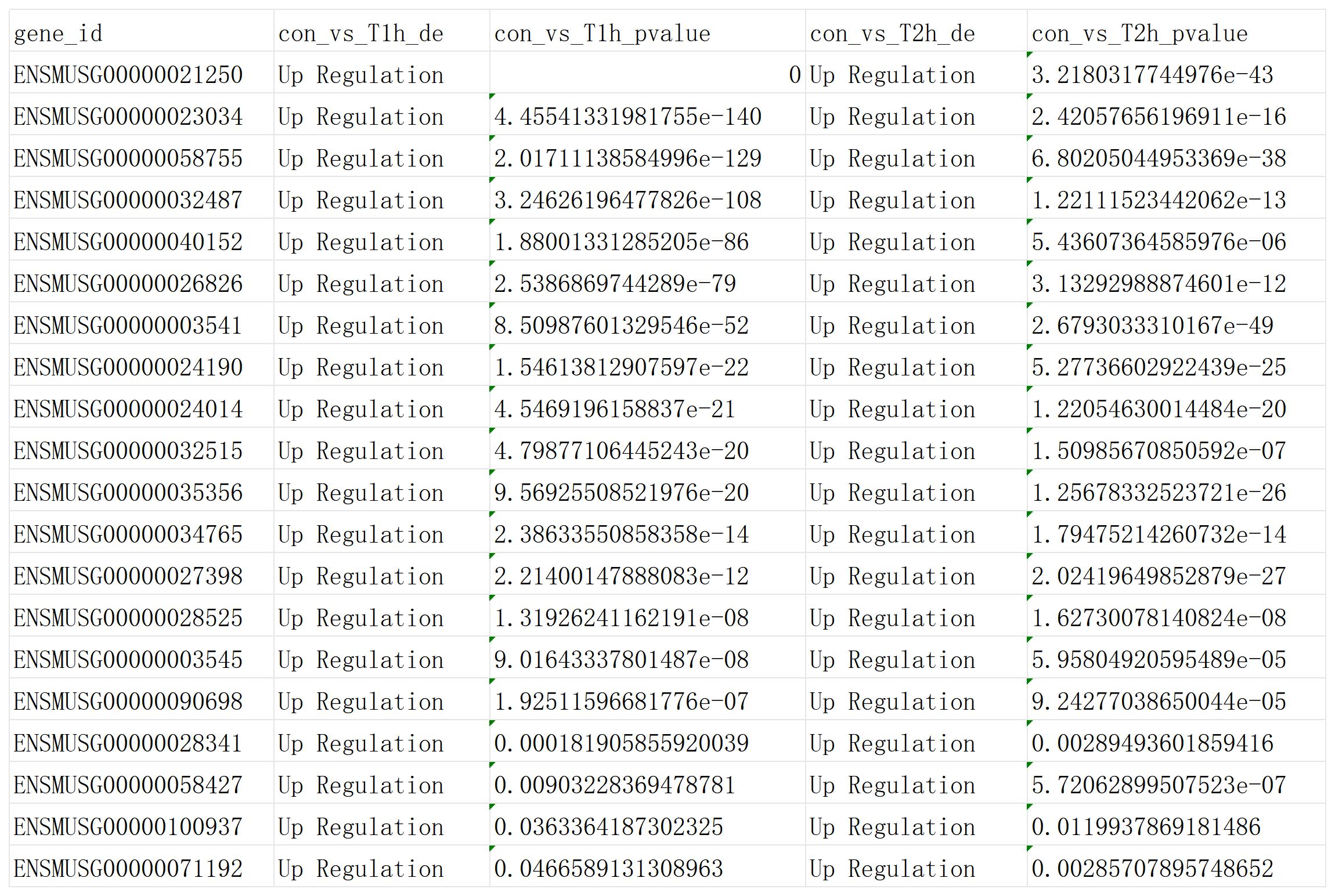


**Supplementary Figure 1. ZVAD does not protect microglia from Bc7-induced cell death.**

**
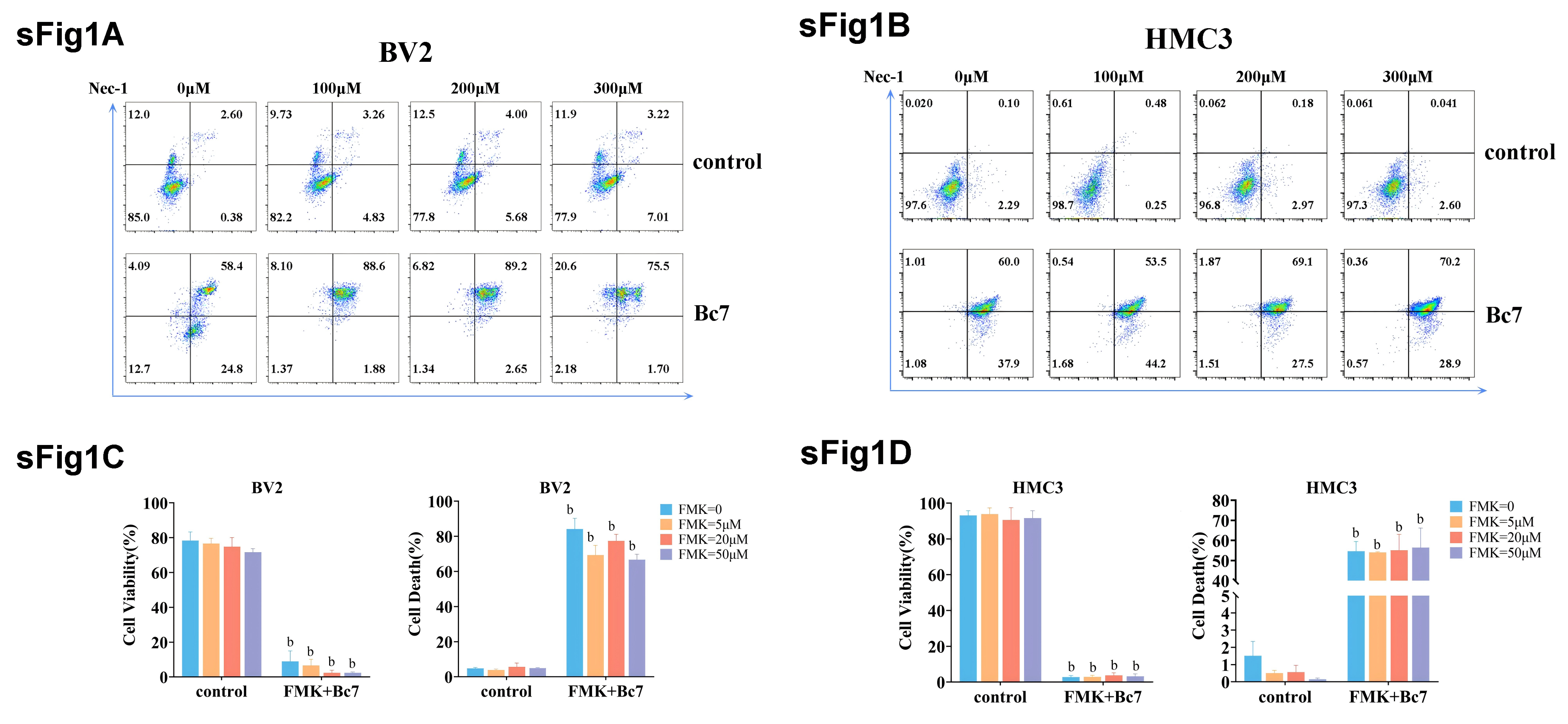
**

(A, B) BV2 (A) and HMC3 (B) cells were infected with Bc7 (MOI = 2, 2 h) with or without ZVAD (FMK) pretreatment at indicated concentrations. Cell death was analyzed by Annexin V/PI staining and flow cytometry. Representative dot plots are shown.

(C, D) Quantification of viable, late apoptotic, and necrotic cells from (A) and (B). Data are shown as mean ± SEM from three independent experiments. ^b^p < 0.0001 vs. the corresponding drug concentration gradient in the control

**Supplementary Figure 2. DSF does not protect microglia from Bc7-induced cell death.**

**
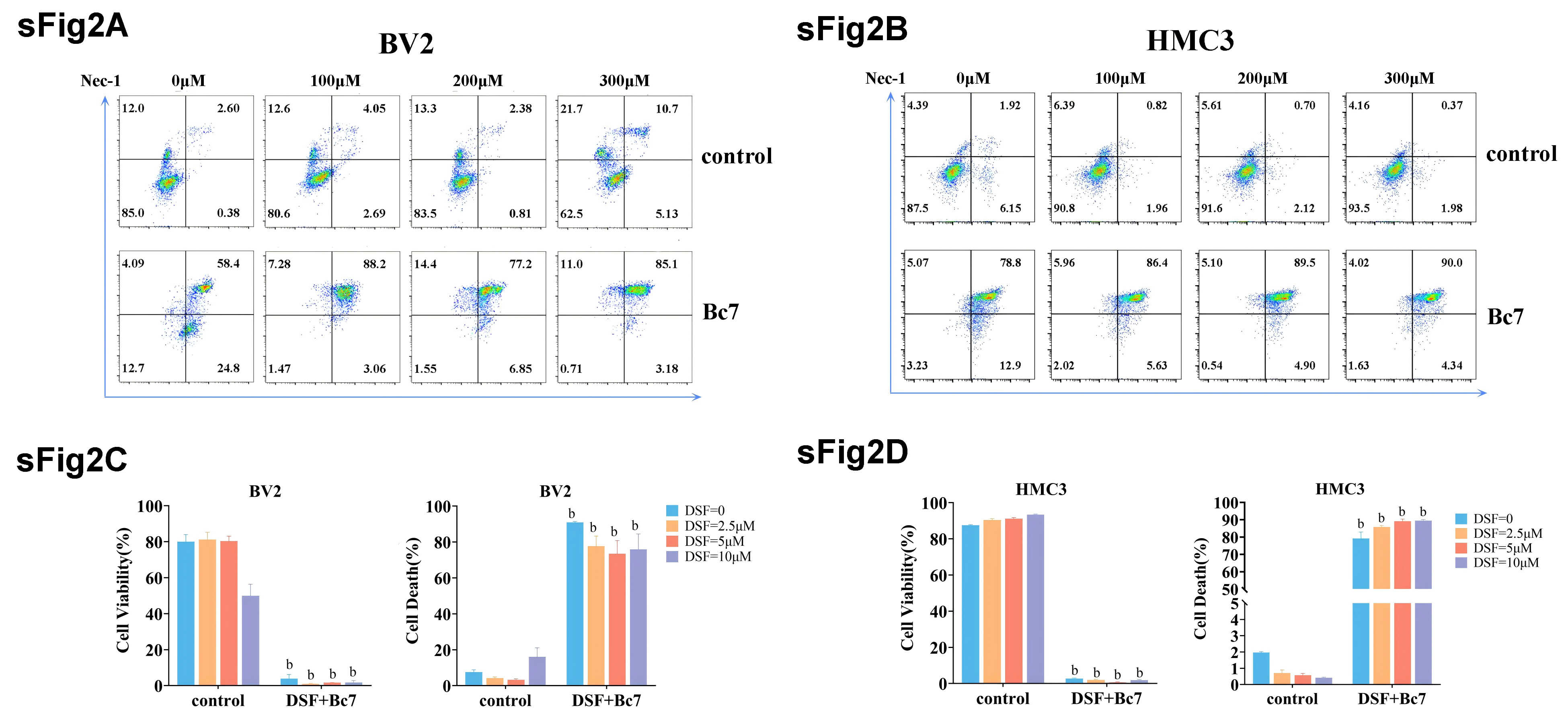
**

(A, B) BV2 (A) and HMC3 (B) cells were infected with Bc7 (MOI = 2, 2 h) with or without DSF pretreatment at indicated concentrations. Cell death was analyzed by Annexin V/PI staining and flow cytometry. Representative dot plots are shown.

(C, D) Quantification of viable, late apoptotic, and necrotic cells from (A) and (B).

Data are shown as mean ± SEM from three independent experiments. ^b^p < 0.0001 vs. the corresponding drug concentration gradient in the control
